# Total and Partial Fat Replacement by Gelled Emulsion (Hemp Oil and Buckwheat Flour) and Its Impact on the Chemical, Technological and Sensory Properties of Frankfurters

**DOI:** 10.3390/foods10081681

**Published:** 2021-07-21

**Authors:** Carmen Botella-Martínez, Manuel Viuda-Martos, José Angel Pérez-Álvarez, Juana Fernández-López

**Affiliations:** IPOA Research Group, Agro-Food Technology Department, Centro de Investigación e Innovación Agroalimentaria y Agroambiental (CIAGRO-UMH), Miguel Hernández University, 03312 Alicante, Spain; c.botella@umh.es (C.B.-M.); mviuda@umh.es (M.V.-M.); ja.perez@umh.es (J.A.P.-Á.)

**Keywords:** hemp oil, fat replacer, buckwheat flour, frankfurter, healthy meat products

## Abstract

A gelled emulsion (GE) prepared with hemp oil and buckwheat flour was used to replace pork back fat in frankfurters. Five different formulations were prepared: control (with 35% pork back fat—SC), and the following four to achieve 25%, 50%, 75%, and 100% pork back fat substitution by GE (S1, S2, S3, and S4, respectively). Nutritional, technological, and sensorial characteristics of frankfurters were evaluated. Sausages containing GE presented a lower total fat content with a higher amount of polyunsaturated fatty acids, increased omega 3 content, and reduced saturated fat by up to 55%. The incorporation of GE did not significantly modify technological properties such as emulsion stability or lipid oxidation in spite of using vegetable oils highly susceptible to oxidation. The reformulation of the frankfurters presented a greater effect on the texture and sensory properties when GE was used as total substitution for the pork back fat (S4). When GE was used only as partial substitution for the pork back fat, sausages similar to control frankfurter were obtained. So this study demonstrated that the use of GE could be a promising strategy in the reformulation of healthier meat products.

## 1. Introduction

Frankfurter is a meat product widely consumed in different regions of the world, mainly attributed to its convenience, low price, nutritional value, and flavor [1,2]. On the other hand, it is well known that a relevant amount of animal fat (pork back fat) is used during its elaboration, reaching in some cases levels close to 40% of fat in the final product [3]. Pork backfat is the most appreciated and valued source of fat in meat products processing mainly due to its chemical composition and saturated fatty acid (SFA) content (approx. 40%) which results not only in technological advantages during processing, but also texture and taste improvement in the final product [4,5,6]. The relationship between saturated fat consumption and certain diseases (mainly obesity and cardiovascular diseases) is moving the meat industry to develop healthier meat products, not only by reducing the amount of fats but also by replacing saturated fats by others with a healthier lipid profile [7,8,9]. Therefore, studies are being carried out in which unsaturated or polyunsaturated oils are used as animal fat replacement in these types of meat product to make them healthier. In any case, this replacement is not an easy task, mainly due to both, technical and sensorial problems in the product, making them difficult to cut and more prone to oxidation [2,4,7]. Within this scene, gelled emulsion (GE) systems have been revealed as a suitable alternative to create animal fat substitutes that provide and improve stability, textural and oxidative properties or without detriment [6,7,10,11,12,13,14,15,16,17,18,19,20,21,22,23]. A GE is a colloidal material in which oil in water emulsions (O/W) coexists within a gel network with mechanical properties similar to a viscoelastic solid [12,13]. For the elaboration of GE, different vegetable oils (chia, hemp, linseed, among others) have been assayed, together with other protein/starchy ingredients such as pseudo-cereal flours (quinoa, amaranth, buckwheat, teff, etc.), with the aim of stabilizing these O/W emulsions [9,12,13,14]. In one of our previous studies, 12 combinations of these vegetable oils and pseudo-cereal flours were used to obtain GE, selecting the combination of hemp oil and buckwheat flour because it was technologically feasible to obtain and showed the best (healthy) fatty acid composition. It presented an interesting contribution of ω-3 and ω-6 fatty acids that could be attractive for reformulating meat products [15,24].

Hemp oil (*Cannabis sativa* L.) is composed of a well-balanced fatty acids and antioxidant profile, is rich in polyunsaturated fatty acids, principally the omega-6 linoleic acid (55.41–56.94%) and contains gamma and alpha linoleic acids (0.64–1.10%, 16.51–20.40%) [25,26]. Buckwheat is a pseudo-cereal with a high nutritional quality known for being a dietary source of dietary fiber, vitamins, minerals, and antioxidants. Its flour contains high levels of essential nutrients and bioactive flavonoids. In reference to that, quercetin and quercetin glycoside, such as rutin and iso-quercetin, have been identified as the major flavonoids in buckwheat, which provide several pharmacological advantages. Many polyphenolics exhibit antioxidative properties, especially oxygen species scavenging [27,28,29,30]. Buckwheat proteins have a high biological value, with a high lysine content, approximately 6 mg/100 g of proteins [31]. The average of protein distribution for buckwheat is 18%–22% of albumin, 15%–70% of globulins, 0%–5% prolamins, and 4%–23% glutens. The protein composition of buckwheat provides emulsifying and gelling properties to buckwheat flour used in the elaboration of the GE [30,32].

No studies have been conducted on the use of this kind of GE in meat products. The objectives of this work were (i) to evaluate the technological viability of producing frankfurters with different pork back fat substitution levels by a GE elaborated with hemp oil and buckwheat flour; (ii) to investigate the effects of different percentages of fat substitution (25%, 50%, 75%, and 100%) on their chemical composition, lipid profile, physico-chemical properties, emulsion and lipid stability, and sensorial properties of frankfurters.

## 2. Materials and Methods

### 2.1. Materials

The ingredients used to make gelled emulsions were: hemp oil (54.44% linoleic acid, 19.95% α-linolenic acid, 8.23% oleic acid, 6.17% palmitic acid, 4.26% linoleic acid, 2.3% stearic acid, 1.62% ϒ-linolenic acid, and 3.03% others, according to the information given by the supplier) from Laboratorios Almond, S.L. (Murcia, Spain); buckwheat flour (BW) distributed by Biogran S.L. (Madrid, Spain); gellan gum (a polysaccharide excreted by microorganism Pseudomonas elodea: this is a water-soluble linear structure with a repeating unit of tetra-saccharide), and instant gel (gelatin from animal origin (pork) with 180 bloom), supplied from Sosa Ingredients S.L. (Barcelona, Spain).

### 2.2. Preparation of Oil-in-Water Gelled Emulsions

Oil-in-water gelled emulsions (GE) were prepared as follows: first the gelling agent “instant gel” was mixed in a homogenizer (Thermomix 31, Vorwerk-España M.S.L., S.C, Spain) with water for 2 min at 60 °C at high speed. Then, the buckwheat flour was added and mixed for 1 min at medium speed. In the next step, the temperature was turned down to 37 °C and “gellan gum” was added and mixed for 2.5 min at 250 rpm. In the last step, the mixture was mixed with the gradual addition of the appropriate amount of hemp oil for 5 min, at 37 °C and 1100 rpm. The GE was then transferred to a refrigerator and stored at 4 °C prior to sausage production. The GE was made combining 47% water, 40% hemp oil, 10% buckwheat flour, 1.5% gellan gum and 1.5% instant gel.

### 2.3. Preparation of Frankfurters

Frankfurter-like sausages were made according to a traditional formula (meat percentages add up to 100% and percentages of others ingredients are related to that of meat): pork lean meat (65%) and pork backfat (35%), 15% water (ice form), 3% potato starch, 1.5% sodium chloride, 300 mg/Kg sodium tripolyphosphate, 150 mg/Kg sodium nitrite, 1.5% casein, 0.2 mL/kg liquid smoke, and spices (a mixture of white pepper and nutmeg).

Five different frankfurter sausage batches (4 kg) were designed to modify lipid concentration, as follows: batch 1 was used as control (SC) with the traditional formula described above. The other four batches were formulated replacing different amounts of animal fat by the GE previously prepared: in the second batch (S1), 25% of pork backfat was replaced by GE; the following batches (S2, S3, and S4) were obtained replacing 50%, 75%, and 100%, respectively, of pork backfat by GE. Three replications of this elaboration process were performed on different days.

The products were prepared in the IPOA Research Group Pilot Plant at the Miguel Hernández University, following an industrial processing protocol. Briefly, meat ingredients were ground in a cutter (1094-Homogeneizer, Tekator, Höganäs, Sweden) and mixed with the sodium chloride and the rest of the ingredients for 2 min (temperature below 12 °C). After homogenization, the resulting meat batter was stuffed using a piston stuffer EM-12 (Mainca, Granollers, Barcelona, Spain) into 20 mm diameter cellulose casings (Fibran, Girona, Spain). Samples were hand linked and cooked in a water bath (80 °C) until the core temperature reached 72 °C. When the endpoint temperature was achieved, the sausages were immediately chilled in ice for 5 min, packed, and stored at 4 °C under darkness conditions.

### 2.4. Emulsion Stability

Emulsion stability of the meat batters (before cooking) was evaluated by means of total expressible fluid (*TEF*) according to Pintado et al. [16] with slight modifications. Samples were centrifugated into centrifuge tubes of 15 mL at 3000 rpm for 1 min. Then they were heated in a water bath at 70 °C for 30 min and cooled at room temperature. Next, they were recentrifuged for 3 min at 3000 rpm. The samples were left standing upside down to release the expressible fluid (fat and water) onto filter paper. The determinations were made in triplicate for each sample. The results are expressed in g of total fluid expelled/100g of sample and were calculated using the following expression (Equation (1)):

(1) $$\%TEF=\frac{\mathrm{Weight}\;\mathrm{of}\;\mathrm{tube}\;\mathrm{with}\;\mathrm{sample}-\mathrm{Weight}\;\mathrm{of}\;\mathrm{tube}\;\mathrm{with}\;\mathrm{pellet}}{\mathrm{Weight}\;\mathrm{of}\;\mathrm{sample}}\times 100.$$

### 2.5. Frankfurters’ Characterization

#### 2.5.1. Proximate Composition

Total moisture, ash, protein, and fat content of the sausages were determined according to the Association of Official Analytical Chemist analysis [33]. The determinations were made in triplicate for each sample.

#### 2.5.2. Fatty Acid Profile

Lipid extraction from the samples was conducted according to Folch et al. [34], and the lipid phase was methylated according to the AOAC [33] method 969.33. The fatty acid methyl esters (FAMEs) were injected into an HP 6890 gas chromatography equipment with a flame ionizer detector and a Suprewax-280 capillary column (30 m, 0.25 μm of film, 0.25 mm internal diameter; Tecknokroma Barcelona, Spain). The temperature program was as follows: the initial temperature was 60 °C for 1 min, then raised at a rate of 10 °C/min until reaching 170 °C and was kept at this temperature for 2 min. Then it was raised at a speed of 3 °C/min until 230 °C for 10 min, and finally, it was raised at a speed of 2 °C/min until 260 °C and maintained for 1 min at this temperature. Helium was the carrier gas with an internal column pressure of 11 psi. The injector volume was 0.2 μL in splitless. The response factors were calculated using fatty acid standards and their identification was made by comparison with the retention times of these FAME standards (Supelco 37 component FAME Mix, Bellefonte, PA, USA). Acetonitrile and formic acid (1%) were used as mobile phase and the flow rate was set at 1 mL/min. All analyzes were carried out in triplicate and the results were expressed as g fatty acid/100 g oil.

Atherogenic index (*AI*) and thrombogenic index (*TI*) were calculated by Equations (2) and (3), respectively, developed by Ulbricht & Southgate [35].

(2) $$AI=\frac{C12:0+\left(4xC14:0\right)+C16:0}{\sum AGMI+\sum n6+\sum n3}$$

(3) $$TI=\frac{C14:0+C16:0+C18:0}{(0,5x\sum MUFA)+(0,5x\sum n6)+(3x\sum n3)+\left(\frac{\sum n3}{\sum n6}\right)}$$

The hypo-cholesterolemic/hypercholesterolemic ratio (h/H) was calculated using Equation (4), (Equation (4)) as described by Fernández et al. [36].

(4) hH=C18:1n9+C18:1n7+∑PUFAC14:0+C16:0

#### 2.5.3. Physico-Chemical Properties

##### pH

The pH of the final products was measured in triplicate with a pH-meter Crison model 510, (Barcelona, Spain) using a penetration probe at different sites of the sample (6 measures).

##### Water Activity

Water activity (aw) was measured at 25 °C using an electrolytic hygrometer (Novasina TH-500, Novasina, Axair Ltd., Pfaeffikon, Switzerland). Three replications of each sample were made.

##### Texture

Texture measurements (texture profile analysis, TPA) were performed using a TA-XT2i Texture Analyzer (Stable Micro Systems, Surrey, England). Sausage sections of 2 cm height in horizontal were submitted to two compression cycles up to 75% at a constant velocity of 1 mm/s at 15–20 °C. From the curves obtained (force-time deformation), the following parameters were calculated: hardness (N), as the maximum peak force during the first compression; adhesiveness (N s), as the negative work between the two compression cycles; springiness (mm), as the height that the food recovers during the time between the end of the first compression and the beginning of the second compression; cohesiveness (dimensionless), as the ratio of the positive force area during the second compression to that during the first compression; and chewiness (N mm), as the product of hardness times, cohesiveness times and springiness [37]. Five measurements per sample were made.

##### Color

The instrumental color parameters were performed directly on the cross-sections of frankfurters using a CM-700 spectrophotometer (Minolta Camera Co., Osaka, Japan), operating with D65 illuminant, 10° observer angle, SCI mode, and with a low reflectance glass (Minolta CR-A51/1829-752) placed between the samples and the equipment. The CIELAB coordinates determined were: L* (lightness), a* (+/− red/green), b* (+/− yellow/blue). Nine readings of each sample were performed at room temperature (25 °C).

The magnitudes h* (hue) and C* (chrome) were calculated with Equations (5) and (6) (Equations (5) and (6)), respectively.

(5) $$C{*}^{\;}=\sqrt{a^{*2}+b^{*2}}$$

(6) $$h*=arctg(b^{*}/a^{*})$$

Total color differences (∆*E*) of each sample (*S*) with respect to control sausage (*C*) were also calculated (Equation (7)).

(7) $$\Delta E=\sqrt{{\left(L_{S}^{*}-L_{CON}^{*}\right)}^{2}+{\left(a_{S}^{*}-a_{CON}^{*}\right)}^{2}+{\left(b_{S}^{*}-b_{CON}^{*}\right)}^{2}}$$

#### 2.5.4. Lipid Oxidation

The TBARs (thio-barbituric acid reactive substances) values were determined in triplicate according to Rosmini et al. [38]. Results, expressed as mg malondialdehyde (MDA)/kg sample, were calculated from a malonaldehyde (MDA) standard curve.

### 2.6. Sensory Assessment

The sensory evaluation was carried out in a sensory analysis laboratory with partitioned cabinets under white lights in individual booths. A 17-member sensory panel aged 18–55 years and with no specific training in the sensory analysis of frankfurters, were recruited from the staff and students at the Miguel Hernández University. The sensory analysis scheme was developed with a hedonic scale consisted of 7 levels (1: dislike extremely and 7: like extremely) on pieces of 2.0 cm approx. thickness (cutting from the frankfurter), evaluating the following attributes: color, hardness, juiciness, hemp smell, hemp flavor, salty flavor, and general acceptability.

### 2.7. Statistical Analysis

From each elaboration process (3), three samples were obtained from each of the five batches (formulation). Means and standard deviations of data obtained from the analysis of frankfurters are shown in corresponding tables and figures. Data were evaluated by one-way analysis of variance (ANOVA); if statistically significant differences were found, a Tukey-b post-hoc test was performed at 5% significance level (*p* < 0.05) using SPSS software (version 24.0, SPSS Inc., Chicago, IL, USA).

## 3. Results & Discussion

### 3.1. Properties of Gelled Emulsion

The total characterization of the GE prepared with hemp oil and buckwheat flour has been previously published [15]. Some of the main properties with special relevance in this study are pH 6.06 ± 0.01, protein content 2.63 ± 0.02 g/100 g of sample, appropriate stability against centrifugation forces (16.83 ± 1.43% TEF) with very little separation between phases after centrifugation. The amount of saturated and unsaturated fatty acids for this GE are 10.24 ± 0.08% and 89.77 ± 0.06%, respectively. The main fatty acids are linoleic acid (54.44%) followed by α-linolenic acid (19.95%), oleic acid (8.23%), and palmitic acid (6.17%). This GE contains approximately 20% of fatty acids n-3.

### 3.2. Emulsion Stability of Meat Batters

**Figure 1:**
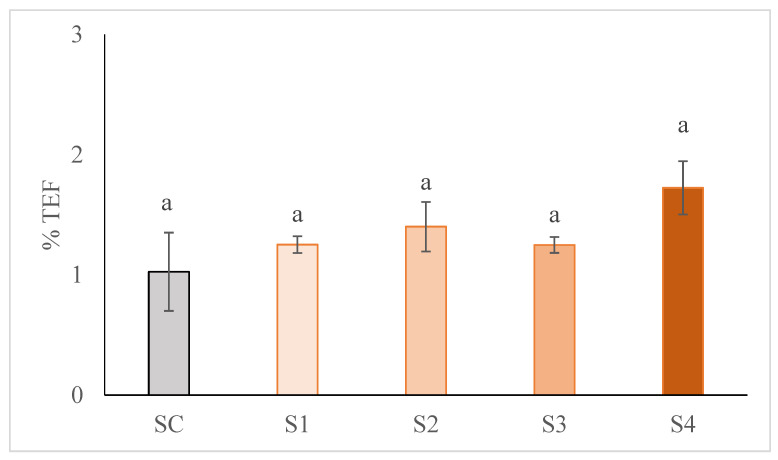
Effect of partial and total replacement of pork back fat by GE, from hemp oil and buckwheat flour, on emulsion stability of frankfurters. Results are expressed as % total expressible fluid (%TEF). SC: frankfurter without GE; S1: frankfurter formulated with GE as 25% fat replacer; S2: frankfurter formulated with GE as 50% fat replacer; S3: frankfurter formulated with GE as 75% fat replacer, and S4: frankfurter formulated with GE as 100% fat replacer. For each parameter, results followed by the same case letter are not significantly different according to Tukey’s HSD post-hoc test (*p*  > 0.05). Data are presented as the mean values of replications ± SD.

The stability of the emulsion was determined on the raw dough before the products were stuffed and cooked and is shown in Figure 1. The higher the TEF percentage, the lower the emulsion stability. The replacement of animal fat by GE (at any percentage) did not cause significant differences (*p* > 0.05) in emulsion stability, values ranging from 1.03 to 1.72 % (Figure 1) in all samples, which is according to normal values for these type of sausages [17]. Some authors have reported that the simple reduction of back fat in cooked sausages decreased their stability [13]. In this case, the water holding capacity of the hydrocolloids present in the GE (gellam gum and instant gel) could be counteracting this negative effect. In addition, it has been reported that the use of a pre-emulsion fat as fat replacement in frankfurters increased their stability reducing the amount of liquid exudate [18,19,39]. In this case, it could be said that all the formulations were stable, as the TEF was low (*p* < 4%) [13].

### 3.3. Properties of Frankfurters Prepared with GE

#### 3.3.1. Proximate Composition

Total moisture, protein, fat, and ash contents of frankfurters are shown in Table 1. The moisture content of sausages ranged from 65.81% to 59.66%, control samples showing the lowest value (*p* < 0.05). The replacement of animal fat by GE increased moisture content without significant differences (*p* > 0.05) between samples with higher substitution percentages (S2 (50% substitution), S3 (75% substitution), and S4 (100% of fat replacement)). A significant reduction in total fat content was achieved in frankfurters as the level of fat replacement (by GE) increased. The decrease in fat contents ranged from 17% to 39%, at different levels of fat replacement. Protein content varied between samples although there was not a clear pattern related to fat replacement: SC and S1 showed the highest protein content and S2 the lowest (*p* < 0.05). No differences were observed in the ash contents regardless of the fat substitution level by GE.

Regarding proximate composition, it is important to highlight that interesting fat reduction levels can be achieved using this GE as fat replacement in frankfurters, which could contribute to decreasing the energy value of this type of meat product, according to new consumer trends. Similar results of fat content reduction have been reported by other authors in different types of meat product (beef patties, hot-dog style sausages or chicken sausages) applying GEs as partial animal fat replacement [21,22,23].

#### 3.3.2. Lipid Profile and Nutritional Parameters

The impact of pork back fat replacement by GE on the lipid profile and nutritional parameters of frankfurters is shown in Table 2. It is known that vegetable oils are rich in unsaturated fatty acids, both monounsaturated (MUFAs) and polyunsaturated (PUFAs). As expected, significant differences (*p* < 0.05) were detected in the fatty acid profile of frankfurters depending on the pork back fat substitution level. In control samples (SC) 21 fatty acids were identified, among which palmitic (C16:0), oleic (C18:1), and linoleic fatty acids (C18:2) make up approximately 80% of the total fat. Together with stearic acid (C18:0), this figure rises to 92% of the total fatty acids, which is in accord with the fatty acid composition of pork back fat reported by other authors [4]. Regarding these four fatty acids, the substitution of pork back fat by GE in frankfurters resulted in a decrease in oleic, palmitic and stearic fatty acids together with an increase in linoleic fatty acid. In addition, to rise tomore than 90% of the total fat, it is necessary to add the amount of other fatty acid, linolenic acid (C18:3). All these changes in fatty acid composition are higher as fat substitution levels increase. At the highest fat substitution level (S4), linoleic acid showed the highest percentage, followed by oleic, and linolenic acid (without differences between them, *p* > 0.05), palmitic acid and stearic acid.

The replacement of animal fat by GE reduced (*p* < 0.05) the amount of saturated fatty acids (SFAs) in frankfurters depending on the substitution level. SC formulated with pork back fat contained the highest amount of SFAs (35.96%), mainly palmitic acid (C16:0) and stearic acid (C18:0). On the other hand, S4 showed the lowest amount of SFAs (16.03%) (palmitic acid and stearic acid) which means a 60% reduction in SFAs. This is due to the acid profile of GE that showed oleic acid (C18:1) as the major MUFA, and linoleic and linolenic acids as the major PUFAs [15]. MUFA content in frankfurters decreased from 50.88% to 17.78% (*p* < 0.05), depending on the fat substitution level. As a result, PUFA content of frankfurters with GE (S1, S2, S3, and S4) increased (*p* < 0.05) as fat substitution level increased. This increase was mainly due to the linoleic acid content that increased from 11.56% (SC) to 45.62% (S4), which means an increase of 75%. In addition, the increase in α- linoleic acid must be noticed (from 0.68% in SC to 15.38% in frankfurters with the highest fat substitution level, S4), which means an increase of 96%. Moreover, according to European regulations [40], S3 and S4 samples could be assigned as sausages with “reduced in saturated fat” as they reached a reduction of more than 25%, while all GE added samples could be labeled with the nutritional claim as “high n-3 fatty acids”, since they contained more than 0.6 g α-linolenic acid per 100 g of the product (1–1.95 g α-linolenic acid per 100 g of frankfurter).

Both the PUFA/SFA and the n−6/n−3 fatty acid ratios are widely studied nutritional parameters because of their association with cardiovascular diseases. It is recommended that the PUFA/SFA ratio be above 0.4 and the n− 6/n −3 ratio below 4 [41]. While all reformulated frankfurters (S1, S2, S3, and S4) fulfilled the PUFA/SFA recommendation, only S2, S3 and S4 samples (50, 75, and 100% pork back fat substitution level, respectively) also fulfilled the n−6/n−3 ratio. By contrast, control sample does not fulfill any requirement regarding these ratios.

The atherogenic index (*AI*) and thrombogenic index (*TI*) are significant parameters to describe possible healthier appeal in meat products [16]. The appearance of various diseases is associated with high values of these indexes [13]. The influence of pork back fat replacement in these parameters was positive considering that both parameters decreased (*p* < 0.05) as pork back fat substitution level increased, indicating a reduction in these cardiovascular risk factors. On the other hand, the hypo-cholesterolemic/hypercholesterolemic index (h/H) also showed a healthy trend depending on the pork back fat substitution level, because it has been described that the better is the highest. As can be seen in Table 2, this index increased as pork back fat substitution level increased, S4 samples (100% substitution) showing the highest value (*p* < 0.05).

#### 3.3.3. pH and Water Activity

pH and water activity values of frankfurters are shown in Table 3. All frankfurters showed pH values in the range considered normal for this type of cooked sausages [2,12,19] with slight differences between some. The substitution of animal fat by GE increased pH values (*p* < 0.05) although this increase was only significant at low substitution level (*p* < 0.05). Several authors have associated higher pH values in cooked sausages with vegetable oil addition, although in other cases this relation was not significant [4]. In spite of the statistical differences between some formulations, these variations do not affect the final quality of the product.

For Aw values, the highest value was found for samples with 100% of fat replacement (S4) and the lowest for control samples (SC) (*p* < 0.05), increasing Aw values depending on the fat substitution level. In any case, the highest Aw value, which was obtained for S4 samples, is in the range of normal Aw values reported for cooked sausages [2,42]. So the pork back fat substitution by GE in frankfurters would not result in a problem related to frankfurter shelf-life.

#### 3.3.4. Color

Color is one of the most studied physical properties in meat products and its objective measurement allows the evaluation of the immediate impact of pork back fat substitution on frankfurters. The color parameters of frankfurters are shown in Table 4. All color parameters were affected (*p* < 0.05) depending on the percentage of fat substitution, all showing a clear behavior (increase or decrease) except for lightness: control frankfurters (SC) and samples with the highest fat substitution level (S4) showed similar (*p* < 0.05) L * values, decreasing toward intermediate pork back fat substitution levels (*p* < 0.05). However, it is important to highlight that the differences showed for L * values between samples were really small (1–2 units) and so without technical importance.

On the other hand, redness values decreased (*p* < 0.05), and yellowness, chroma, and hue values increased (*p* < 0.05) as fat substitution levels increase. This behavior has been previously reported in cooked sausages due to the replacement of pork back fat by several vegetable oils [4,16,19]. The pigment content in these vegetable oils has been attributed as the main reason for this. As the substitution percentage increases, the contribution of red pigments (myoglobin and other derivatives) to the final color decreases, resulting in lower a * and higher b * values in frankfurters. In addition, it has been reported that hemp oil has a high content in total chlorophylls (up to 57.66 mg/kg) and carotenes (up to 146.80 mg/kg) [43], and both pigments would be responsible for this increase in b * values. In this case, it is also clear that the behavior of color saturation (C *) is dependent only on the b * evolution.

Nevertheless, taking into consideration the color differences (Δ*E* *) with respect to control frankfurter (SC), it could be said that all show differences easily detected by the human eye (>3 units; Martínez et al. [44]). These color differences would be expected because of the GE color, which could be defined as greenish and yellowish, mainly because of the color of the hemp oil and buckwheat flour. In any case, the color of the frankfurters with GE, although different from the control, is still a typical color of frankfurter-like sausages, for example of the Bratwurst type, and so it should not be a reason to reject them.

#### 3.3.5. Texture

Texture is a considerable factor for consumers of meat products and one of the quality parameters generally most affected due to the replacement of pork back fat (solid) by vegetal oils (liquid). To avoid this problem, GE has been developed in order to retain solid-like properties allowing oil stabilization and structuring [14,15,20,24]. The results of texture profile analysis are given in Table 5. Adhesiveness, cohesiveness, and chewiness did not differ between formulations (*p* > 0.05). Hardness and springiness were affected (*p* < 0.05) by the substitution of pork back fat by GE. In the case of hardness, this effect was only significant at the highest substitution level (S4; 100%) which showed the lowest value (*p* < 0.05). This could be related to the chemical composition because increased moisture together with decreased fat makes the final product less dense [45]. Only springiness in S2 sample (0.34 ± 0.02) differed (*p* < 0.05) from the control (0.26 ± 0.02) and, although the result was significant, quantitatively this effect was very small. Controversial results in textural properties have been reported in cooked sausages due to the partial/total pork back fat replacement by GEs. In most of the cases hardness was the property most affected, but sometimes this was greater and at other times reduced [10,11,13,14]. So it could be concluded that differences in texture properties may be related to the specific characteristics of each GE and its impact in the meat matrix.

#### 3.3.6. Lipid Oxidation

**Figure 2:**
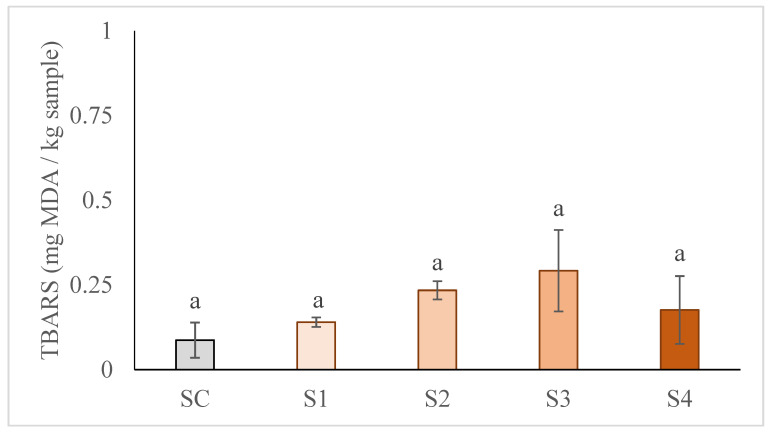
Effect of partial and total replacement of pork back fat by the gelled emulsion, from hemp oil and buckwheat flour, on lipid oxidation of frankfurters. SC: frankfurter without GE; S1: frankfurter formulated with GE as 25% fat replacer; S2: frankfurter formulated with GE as 50% fat replacer; S3: frankfurter formulated with GE as 75% fat replacer, and S4: frankfurter formulated with GE as 100% fat replacer. For each parameter, results followed by the same case letter are not significantly different according to Tukey’s HSD post-hoc test (*p* > 0.05). Data are presented as the mean values of replications ± SD.

As can be seen in Figure 2, all samples showed a low lipid oxidation level, without differences between them (*p* > 0.05). All samples showed TBARS values ranging from 0.09 to 0.29 mg MA/ Kg of sample, lower than the rancidity detection limit (>1.0 mg MA/Kg of sample, Verna & Sahoo [46]). This is a very positive result taking into account that vegetable oils are more susceptible to lipid oxidation, due to their higher PUFA content. Therefore, in the case of these reformulated frankfurters (S1, S2, S3, and S4), although the amount of PUFA has increased (Table 2), this has not increased their lipid oxidation. Several authors have attributed this to the capsulated oil droplets in the gel matrix, which would act as a protective barrier against oxidation [39]. According to this positive result, even a total replacement of pork back fat by this GE would not cause a significant increase in oxidation. However, a shelf-life study would be necessary to confirm this behavior in reformulated frankfurters. Alejandre et al. [22] reported a similar trend for lipid oxidation in beef patties in which fat was totally replaced with a gel emulsion containing microalgal oil and blackthorn branch extract. They also suggested that the lower fat content of the reformulated patties could explain this lipid oxidation control.

### 3.4. Sensory Evaluation

**Figure 3:**
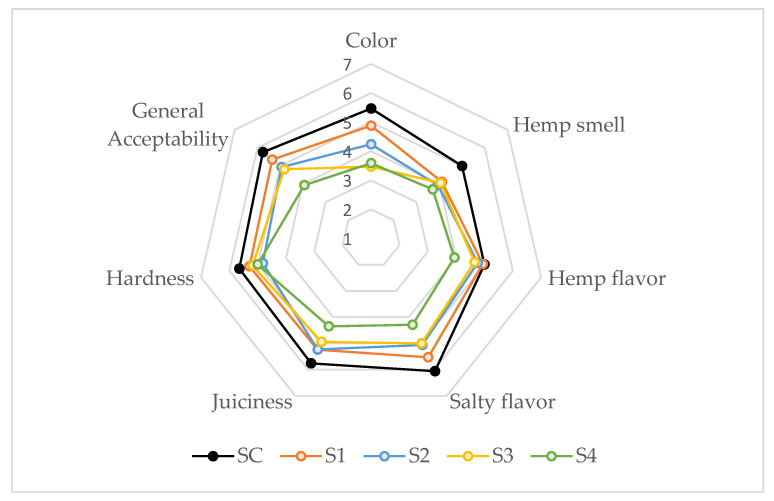
Effect of partial and total replacement of pork back fat by the gelled emulsion, from hemp oil and buckwheat flour, on sensory parameters. SC: frankfurter without GE; S1: frankfurter formulated with GE as 25% fat replacer; S2: frankfurter formulated with GE as 50% fat replacer; S3: frankfurter formulated with GE as 75% fat replacer, and S4: frankfurter formulated with GE as 100% fat replacer.

Sensory quality should be evaluated because the modification of fatty acids in meat products is a challenging procedure. Figure 3 shows the results of the sensory quality of lipid-modified samples for color, hemp smell, hemp flavor, salty flavor, juiciness, hardness, and acceptability. Hemp flavor, juiciness, and hardness scores were similar (*p* > 0.05) for all samples. Hemp smell and salty flavor scores followed the same pattern: only S4 samples scored significantly lower (*p* < 0.05) than control. Color was the attribute influenced mostly by frankfurter reformulation; in this case, S3 and S4 samples presented lower scores (*p* < 0.05) than control and S1. The color score of S3 and S4 were significantly lower (*p* < 0.05) than control and S1 samples. So# it seems clear that the inclusion of GE (at high percentages) altered visual sensory features, which could be due to the color of the hemp oil as has been discussed in Section 3.3.4. The color and hemp smell (scores <4) are the main reasons for the lower sensory acceptance of the frankfurters with high GE substitution percentages (S4, 100%). Other researchers using GE as partial and total substitutes for pork back fat in cooked sausages also verified that color and flavor were the attributes most rejected by consumers [47]. Most authors reported unpleasant sensory characteristics in different types of meat products in which the animal fat replacement by GE was made at high percentages [13,19,21].

## 4. Conclusions

This research suggests that the reformulation of cooked sausages (frankfurters) using hemp oil and buckwheat flour as a gelled emulsion as pork back fat substitute is feasible and represents a viable alternative for improving nutritional composition, without adversely affecting either the technological properties or the typical appearance of the resulting product, mainly when this gelled emulsion was used as partial replacement of pork back fat. A reduction of 17%–39% of total fat was obtained with an improved lipid profile (reduction in saturated and increase in polyunsaturated fatty acids). In general, although sensorial differences were detected for frankfurters in which pork back fat was totally replaced by this GE (S4), all the others were evaluated as acceptable by panelists. However, even in the case of a total replacement, the sensory limitations in terms of flavor negative attributes could be easily overcome by reformulating the product with other spice mixtures or increasing its smoked flavor. Other than the quality aspects, these reformulation strategies did not result in higher lipid oxidation, despite the substitution by more susceptible oils. In any case, shelf-life studies would be necessary to confirm these results in reformulated frankfurters, and to assess their stability and microbiological quality during storage.

## Figures and Tables

**Table 1:** Effect of partial and total replacement of pork back fat by the gelled emulsion from hemp oil and buckwheat flour, on proximate composition of frankfurters.

Sample	Moisture	Ash	Fat	Protein
SC	59.66 ± 0.03 ^d^	2.34 ± 0.15 ^a^	20.75 ± 0.28 ^a^	14.59 ± 0.25 ^a^
S1	61.65 ± 0.56 ^c^	2.15 ± 0.07 ^a^	17.20 ± 0.32 ^b^	14.17 ± 0.19 ^a,b^
S2	64.94 ± 0.03 ^a,b^	2.14 ± 0.14 ^a^	17.02 ± 0.63 ^b^	12.61 ± 0.10 ^d^
S3	64.87 ± 0.10 ^b^	3.11 ± 1.47 ^a^	14.78 ± 0.09 ^c^	13.48 ± 0.22 ^bc^
S4	65.81 ± 0.02 ^a^	2.39 ± 0.04 ^a^	12.69 ± 0.10 ^d^	13.41 ± 0.15 ^c^

Results are expressed as g/100 g. SC: frankfurter without GE; S1: frankfurter formulated with GE as 25% fat replacer; S2: frankfurter formulated with GE as 50% fat replacer; S3: frankfurter formulated with GE as 75% fat replacer, and S4: frankfurter formulated with GE as 100% fat replacer. For each parameter, results followed by the same case letter (a–d) are not significantly different according to Tukey’s HSD post-hoc test (*p* > 0.05). Data are presented as the mean values of replications ± SD.

**Table 2:** Effect of partial and total replacement of pork back fat by the gelled emulsion from hemp oil and buckwheat flour, on lipid profile and nutritional parameters of frankfurters.

Fatty Acids (%)	SC	S1	S2	S3	S4
C6:0	0.01 ± 0.00 ^aL^	0.01 ± 0.00 ^aM^	0.02 ± 0.00 ^aM^	0.02 ± 0.00 ^aN^	0.02 ± 0.00 ^aN^
C8:0	0.02 ± 0.00 ^aL^	0.03 ± 0.00 ^aM^	0.03 ± 0.00 ^aM^	0.04 ± 0.00 ^aN^	0.03 ± 0.00 ^aN^
C10:0	0.06 ± 0.01 ^aK^	0.06 ± 0.01 ^aL^	0.05 ± 0.00 ^bL^	0.04 ± 0.00 ^cN^	0.02 ± 0.00 ^dN^
C12:0	0.08 ± 0.00 ^aK^	0.07 ± 0.00 ^aL^	0.06 ± 0.00 ^bL^	0.05 ± 0.00 ^cN^	0.02 ± 0.00 ^dN^
C13:0	ND	ND	ND	ND	0.02 ± 0.00 ^N^
C14:0	1.27 ± 0.02 ^aE^	1.13 ± 0.02 ^bG^	0.95 ± 0.02 ^cG^	0.67 ± 0.01 ^dJ^	0.37 ± 0.00 ^eJ^
C15:0	0.07 ± 0.00 ^aK^	0.06 ± 0.01 ^aL^	0.05 ± 0.01 ^bL^	0.05 ± 0.00 ^bN^	0.03 ± 0.00 ^cN^
C16:0	22.92 ± 0.03 ^aB^	21.10 ± 0.05 ^bB^	18.26 ± 0.21 ^cC^	14.39 ± 0.09 ^dC^	9.96 ± 0.13 ^eC^
C16:1trans	0.42 ± 0.00 ^aG^	0.35 ± 0.01 ^bJ^	0.26 ± 0.01 ^cJ^	0.22 ± 0.00 ^dL^	0.14 ± 0.00 ^eL^
C16:1cis	2.21 ± 0.01 ^aD^	1.96 ± 0.05 ^bF^	1.53 ± 0.01 ^cF^	1.04 ± 0.00 ^dH^	0.54 ± 0.01 ^eI^
C17:0	0.38 ± 0.01 ^aG^	0.36 ± 0.01 ^bJ^	0.31 ± 0.00 ^cJ^	0.25 ± 0.00 ^dL^	0.17 ± 0.00 ^eL^
C17:1	0.34 ± 0.00 ^aH^	0.31 ± 0.00 ^aJ^	0.25 ± 0.02 ^abJ^	0.19 ± 0.00 ^bM^	0.07 ± 0.04 ^cM^
C18:0	10.94 ± 0.04 ^aC^	10.36 ± 0.03 ^bD^	9.47 ± 0.02 ^cD^	7.07 ± 0.02 ^dE^	4.44 ± 0.07 ^eD^
C18:1 *cis*	46.71 ± 0.10 ^aA^	40.45 ± 0.55 ^bA^	32.38 ± 0.08 ^cA^	23.53 ± 0.10 ^dB^	15.56 ± 0.44 ^eB^
C18:1 *trans*	0.10 ± 0.00 ^bJ^	0.09 ± 0.00 ^bL^	0.07 ± 0.00 ^bL^	1.34 ± 0.00 ^aG^	0.92 ± 0.16 ^aG^
C18:2 (*n-6*)	11.56 ± 0.01 ^eC^	17.51 ± 0.38 ^dC^	25.54 ± 0.13 ^cB^	35.09 ± 0.01 ^bA^	45.62 ± 0.06 ^aA^
C18:2(*n-3*)	0.09 ± 0.01 ^eJ^	0.65 ± 0.04 ^dH^	1.45 ± 0.02 ^cF^	2.31 ± 0.00 ^bF^	3.30 ± 0.00 ^aE^
C18:3 (*n-3*)	0.68 ± 0.01 ^eF^	3.29 ± 0.18 ^dE^	6.79 ± 0.17 ^cE^	10.81 ± 0.00 ^bD^	15.38 ± 0.00 ^aB^
C18:3 (*n-6*)	ND	0.22 ± 0.02 ^dK^	0.50 ± 0.03 ^cH^	0.84 ± 0.00 ^bI^	1.22 ± 0.01 ^aF^
C20:0	0.20 ± 0.00 ^eI^	0.31 ± 0.00 ^dJ^	0.46 ± 0.01 ^cH^	0.61 ± 0.00 ^bJ^	0.75 ± 0.02 ^aH^
C20:1	1.10 ± 0.03 ^aE^	0.95 ± 0.01 ^bG^	0.84 ± 0.01 ^cG^	0.67 ± 0.00 ^dJ^	0.55 ± 0.00 ^eI^
C20:2	0.54 ± 0.01 ^aF^	0.47 ± 0.00 ^bI^	0.40 ± 0.01 ^cI^	0.32 ± 0.00 ^dK^	0.24 ± 0.01 ^eK^
C20:3	0.31 ± 0.01 ^aH^	0.30 ± 0.00 ^abJ^	0.27 ± 0.00 ^abJ^	0.25 ± 0.01 ^bcL^	0.20 ± 0.01 ^cK^
C20:5	ND	ND	0.15 ± 0.00 ^cK^	0.24 ± 0.00 ^bL^	0.32 ± 0.01 ^aJ^
C24:0	ND	ND	ND	ND	0.20 ± 0.03 ^K^
Σ *n-3*	0.98 ± 0.02 ^eG^	3.59 ± 0.03 ^dG^	7.21 ± 0.07 ^cD^	11.30 ± 0.17 ^bE^	15.90 ± 0.07 ^aD^
Σ *n-6*	12.19 ± 0.05 ^dE^	18.85 ± 0.08 ^dE^	27.89 ± 0.11 ^cC^	38.56 ± 0.01 ^bC^	50.39 ± 0.05 ^aC^
*n-6/n-3* ratio	12.39 ± 0.01 ^aE^	5.25 ± 0.02 ^bF^	3.87 ± 0.01 ^cE^	3.41 ± 0.03 ^dG^	3.17 ± 0.05 ^eF^
Σ SFA	35.96 ± 0.04 ^aC^	33.49 ± 0.06 ^bC^	29.66 ± 0.01 ^cC^	23.17 ± 0.00 ^dD^	16.03 ± 0.01 ^eD^
Σ UFA	64.05 ± 0.03 ^eA^	66.55 ± 0.21 ^dA^	70.42 ± 0.24 ^cA^	76.85 ± 0.07 ^bA^	84.06 ± 0.22 ^aA^
Σ MUFA	50.88 ± 0.02 ^aB^	44.10 ± 0.04 ^bB^	35.33 ± 0.09 ^cB^	26.98 ± 0.04 ^dD^	17.78 ± 0.18 ^eD^
Σ PUFA	13.17 ± 0.01 ^eD^	22.44 ± 0.02 ^dD^	35.10 ± 0.06 ^cB^	49.87 ± 0.02 ^bB^	66.28 ± 0.13 ^aB^
Σ PUFA/ Σ SFA	0.37 ± 0.01 ^eH^	0.67 ± 0.03 ^dI^	1.18 ± 0.02 ^cF^	2.15 ± 0.07 ^bG^	4.13 ± 0.02 ^aF^
AI *	0.44 ± 0.01 ^aH^	0.39 ± 0.00 ^bJ^	0.31 ± 0.01 ^cG^	0.22 ± 0.00 ^dH^	0.14 ± 0.00 ^eG^
TI *	1.02 ± 0.04 ^aG^	0.77 ± 0.01 ^bI^	0.54 ± 0.01 ^cG^	0.33 ± 0.00 ^dH^	0.18 ± 0.00 ^eG^
h/H *	2.48 ± 0.03 ^eF^	2.83 ± 0.04 ^dH^	3.52 ± 0.04 ^cE^	4.96 ± 0.02 ^bF^	8.01 ± 0.07 ^aE^

Results are expressed as g/100g. ND: not detected. SFA: saturated fatty acids; UFA: unsaturated fatty acids; MUFA: monounsaturated fatty acids; PUFA: Polyunsaturated fatty acids. * AI (atherogenic index), TI (thrombogenic index), h/H (hypo-cholesterolemic/hypercholesterolemic index). A lower-case letter (a–e) refers to the comparison of the same fatty acid between the different oil samples, while an upper-case letter (A–N) refers to the comparison of the different fatty acids in the same sample; results followed by the same lower/upper-case letter are not significantly different according to Tukey’s HSD post-hoc test (*p* > 0.05). Data are presented as the mean values of replications ± SD.

**Table 3:** Effect of partial and total replacement of pork back fat by the gelled emulsion, from hemp oil and buckwheat flour, on pH and water capacity of frankfurters.

Sample	pH	Aw
SC	5.88 ± 0.01 ^c^	0.947 ± 0.003 ^c^
S1	5.94 ± 0.01 ^b^	0.953 ± 0.001 ^bc^
S2	6.09 ± 0.01 ^a^	0.956 ± 0.002 ^bc^
S3	5.91 ± 0.01 ^bc^	0.960 ± 0.003 ^b^
S4	5.90 ± 0.01 ^c^	0.969 ± 0.001 ^a^

SC: frankfurter without GE; S1: frankfurter formulated with GE as 25% fat replacer; S2: frankfurter formulated with GE as 50% fat replacer; S3: frankfurter formulated with GE as 75% fat replacer, and S4: frankfurter formulated with GE as 100% fat replacer. For each parameter, results followed by the same case letter (a–c) are not significantly different according to Tukey’s HSD post-hoc test (*p* > 0.05). Data are presented as the mean values of replications ± SD.

**Table 4:** Effect of partial and total replacement of pork back fat by the gelled emulsion from hemp oil and buckwheat flour on color parameters of frankfurters.

Sample	L *	a *	b *	C *	h	Δ*E* *
SC	72.7 ± 0.63 ^a^	3.42 ± 0.35 ^a^	9.15 ± 0.24 ^e^	9.77 ± 0.30 ^e^	69.53 ± 1.71 ^e^	-
S1	70.42 ± 0.87 ^bc^	2.88 ± 0.19 ^b^	11.32 ± 0.42 ^d^	11.68 ± 0.44 ^d^	75.70 ± 0.58 ^d^	3.30 ± 0.47 ^d^
S2	69.53 ± 1.29 ^c^	2.80 ± 0.25 ^b^	13.06 ± 1.72 ^c^	13.36 ± 1.71 ^c^	77.72 ± 1.46 ^c^	5.36 ± 1.27 ^c^
S3	70.99 ± 0.90 ^b^	1.21 ± 0.47 ^c^	15.23 ± 0.31 ^b^	15.29 ± 0.30 ^b^	85.45 ± 1.79 ^b^	6.77 ± 0.24 ^b^
S4	72.18 ± 0.27 ^a^	0.38 ± 0.16 ^d^	17.01 ± 0.33 ^a^	17.01 ± 0.33 ^a^	88.73 ± 0.54 ^a^	8.45 ± 0.33 ^a^

SC: frankfurter without GE; S1: frankfurter formulated with GE as 25% fat replacer; S2: frankfurter formulated with GE as 50% fat replacer; S3: frankfurter formulated with GE as 75% fat replacer, and S4: frankfurter formulated with GE as 100% fat replacer. For each parameter, results followed by the same case letter (a–e) are not significantly different according to Tukey’s HSD post-hoc test (*p* > 0.05). Data are presented as the mean values of replications ± SD.

**Table 5:** Effect of partial and total replacement of pork back fat by the gelled emulsion from hemp oil and buckwheat flour, on texture profile of frankfurters.

Sample	Hardness (N)	Adhesiveness (N s)	Springiness (mm)	Cohesiveness	Chewiness (N mm)
SC	87.11 ± 8.86 ^ab^	−0.67 ± 0.34 ^a^	0.26 ± 0.02 ^b^	0.78 ± 0.04 ^a^	17.36 ± 2.12 ^a^
S1	93.51 ± 9.73 ^a^	−0.72 ± 0.36 ^a^	0.29 ± 0.04 ^ab^	0.75 ± 0.03 ^a^	20.83 ± 4.45 ^a^
S2	83.91 ± 11.22 ^ab^	−0.52 ± 0.32 ^a^	0.34 ± 0.02 ^a^	0.75 ± 0.02 ^a^	21.37 ± 3.71 ^a^
S3	89.86 ± 20.73 ^ab^	−0.49 ± 0.32 ^a^	0.33 ± 0.05 ^ab^	0.76 ± 0.03 ^a^	22.82 ± 7.66 ^a^
S4	63.22 ± 9.72 ^b^	−0.91 ± 0.34 ^a^	0.25 ± 0.04 ^b^	0.81 ± 0.02 ^a^	12.92 ± 3.27 ^a^

SC: frankfurter without GE; S1: frankfurter formulated with GE as 25% fat replacer; S2: frankfurter formulated with GE as 50% fat replacer; S3: frankfurter formulated with GE as 75% fat replacer, and S4: frankfurter formulated with GE as 100% fat replacer. For each parameter, results followed by the same case letter (a,b) are not significantly different according to Tukey’s HSD post-hoc test (*p* > 0.05). Data are presented as the mean values of replications ± SD.

## Data Availability

Data presented in this study are available on request from the corresponding author.
